# Intermittent Administration of Helminth-Derived Fh15 Modulates Gut Microbiota and Partially Mitigates Dysbiosis in Early Stages of Severe Experimental Colitis

**DOI:** 10.3390/ijms27094068

**Published:** 2026-05-02

**Authors:** María Del Mar Figueroa-Gispert, Natalie M. Meléndez-Vázquez, Ana M. Espino, Filipa Godoy-Vitorino

**Affiliations:** Department of Microbiology and Immunology, University of Puerto Rico-Medical Sciences Campus, San Juan, PR 00936, USA; maria.figueroa14@upr.edu (M.D.M.F.-G.); natalie.melendez2@upr.edu (N.M.M.-V.)

**Keywords:** ulcerative colitis, inflammatory bowel disease, DSS, dysbiosis, helminth, *Fasciola hepatica*, Fh15, gut microbiota

## Abstract

Ulcerative colitis (UC) is a chronic inflammatory bowel disease characterized by dysbiosis of the gut microbiota. Helminth infections are known to modulate host immunity and intestinal microbial composition; however, the therapeutic use of live parasites poses safety challenges. The recombinant *Fasciola hepatica* fatty acid-binding protein Fh15 is a helminth-derived molecule with anti-inflammatory effects in models of septic shock and dextran sulfate sodium (DSS)-induced colitis. Whether Fh15 also influences gut microbial composition during colitis remains unknown. Male C57BL/6 mice received 4% DSS in drinking water for 7 days to induce colitis and were treated intraperitoneally with Fh15 (2 mg/kg) on days 1, 3, and 5. Fecal samples were collected on days 2, 4, and 7 for 16S rRNA gene sequencing. Standard microbiota pipelines were used to evaluate community diversity. Acute DSS treatment disrupted gut microbial diversity and community structure compared with non-colitic controls. Fh15 treatment partially restored early microbial balance by shifting microbial composition toward that of healthy mice and reducing microbial dispersion, indicating enhanced community stability despite severe dysbiosis. Although alpha diversity did not return to control levels, Fh15 mitigated the expansion of pro-inflammatory genera (*Enterococcus* and *Turicibacter*) and preserved beneficial taxa, including *Adlercreutzia*.

## 1. Introduction

Ulcerative colitis (UC) is a chronic inflammatory disease of the colon characterized by relapsing inflammation of the intestinal mucosa [1]. Its etiology is multifactorial, involving complex interactions between genetic predisposition, immune dysregulation, environmental triggers, and alterations in the gut microbiota [2]. Among these, increasing evidence supports a pivotal role for gut microbial imbalance, or dysbiosis, in the pathophysiology of UC [3,4]. Changes in the composition and function of the intestinal microbiota have been associated with disease onset, namely a reduction in bacterial diversity, reshaping of the community structure, and loss of beneficial taxa [5]; progression, including an increase of pathobionts [6]; and relapse [7]. Beyond descriptive human cohort studies using next-generation sequencing (NGS) technologies, much of our understanding of pathogenesis and dysbiosis comes from UC animal models, including those induced by dextran sulfate sodium (DSS) [8], acetic acid [9], genetic modifications [10], and spontaneous colitis [11]. Mice with DSS-induced UC present reductions in Bacillota and Bacteroidota, with increases in Pseudomonadota [12,13]. These shifts are associated with elevated levels of pro-inflammatory cytokines such as TNF-α, IFN-γ, and IL-4. Additionally, *Clostridium sensu stricto*, in other cases, has been linked to exacerbation of inflammation via IL-4, TNF-α, and IFN-γ production [14]. In contrast, the presence of specific beneficial microbes has been negatively correlated with inflammation. Notably, *Faecalibacterium* has been negatively associated with increased levels of IL-6, proposing butyrate production by this genus as a solution for UC pathologies [15]. Similarly, increased abundance of *Bifidobacterium adolescentis* correlates with lower TNF-α expression, suggesting a protective role in maintaining mucosal homeostasis [16]. These findings support the concept that microbiota modulation may serve as a therapeutic approach for UC. The restoration of the gut microbial homeostasis may indeed contribute to disease control, as the overgrowth of opportunistic bacteria contribute to the development of inflammatory diseases such as UC [3,4]. In both human and mouse models of UC, richness and evenness is reduced, while community composition differs significantly from that of healthy individuals [5], indicative of a more dysregulated microbial ecosystem. These alterations reflect decreased microbial resilience, which is often linked to inflammation and disease progression.

Helminths have long co-evolved with both the mammalian immune system and intestinal microbiota [17], resulting in intricate host–parasite–microbe interactions that can profoundly affect host health. These organisms are known to modulate the immune system [18], typically inducing regulatory and anti-inflammatory responses that can counteract excessive inflammation. In recent years, helminth-derived molecules have emerged as promising strategies to modulate the immune response and mitigate inflammation in UC [19]. Several studies have also demonstrated that helminth infections can reshape the gut microbiome [20], often promoting the growth of beneficial bacterial taxa such as *Lactobacillus* while increasing the production of anti-inflammatory metabolites [20,21], including short-chain fatty acids, key indicators of a healthy intestinal environment. Despite the potential benefits, the deliberate reintroduction of live helminths as a therapeutic strategy for autoimmune and inflammatory disorders poses significant safety and ethical challenges. Consequently, recent research has shifted toward identifying and characterizing helminth-derived molecules [22] capable of replicating the immunomodulatory and microbiome-modifying effects of whole-parasite exposure, but in a safer, more controlled manner. To our knowledge, there is only one study that has examined the alleviation or restoration of microbial dysbiosis through a helminth-derived protein administered as a biotherapeutic agent in place of natural or artificial infection [22]. That study found that recombinant *Trichinella spiralis* galectin (rTs-gal) treatment mitigated DSS-induced dysbiosis by decreasing harmful bacteria such as *Helicobacter* and increasing Muribaculaceae and *Ligilactobacillus* probiotic genera that have been demonstrated to inhibit inflammation. At the same time, rTs-gal promoted a partial restoration of gut microbial balance, supporting its protective role in colitis [22].

Another helminth-derived molecule that could potentially remodel the gut microbiota, Fh15, a recombinant fatty acid-binding protein derived from *Fasciola hepatica*, has been widely investigated for its immunomodulatory properties. Fh15 has been shown to exert potent anti-inflammatory effects and to act as a promising biotherapeutic against sepsis in both murine and non-human primate models [23,24]. More recently, in our previous work we demonstrated that Fh15 also displays strong anti-inflammatory properties in a murine model of DSS-induced colitis [25]. That study demonstrated that intraperitoneal administration of Fh15 (2 mg/kg), administered three times per week, mitigates UC severity, alleviates epithelial damage, downregulates pro-inflammatory cytokines, reduces leukocyte infiltration in the distal colon, and suppresses serum levels of myeloperoxidase (MPO) and chitinase-3-like protein 1 (CHI3L1), key inflammatory markers associated with colitis [25]. Given the strong interplay between the gut microbiota and the immune system, evaluating Fh15’s ability to restore DSS-induced microbial dysbiosis is essential to understand its therapeutic potential. We hypothesize that Fh15 treatment modulates gut microbiota dynamics during DSS-induced colitis, reducing dysbiosis and promoting a more stable microbial community. Here we examined the effect of Fh15 on the gut microbiota of DSS-induced colitic mice. Using longitudinal 16S rRNA sequencing at baseline and on days 2, 4, and 7 of treatment, this study provides key insights into how helminth-derived molecules, such as Fh15, may modulate microbial composition and improve gut health in an experimental colitis mouse model.

## 2. Results

### 2.1. Effect of Fh15 on Gut Microbial Community Structure and Diversity in DSS-Induced Ulcerative Colitis

**Figure 1:**
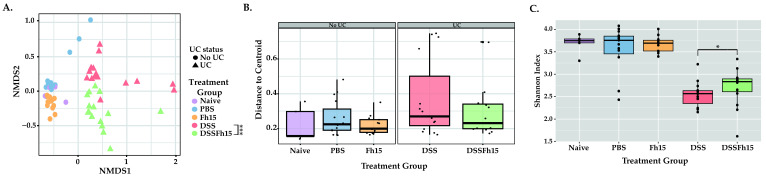
Fh15 treatment partially restores diversity and community structure while shifting the taxonomic composition of ulcerative colitis mice gut microbiota. (**A**) Beta diversity analysis using non-metric multidimensional scaling (NMDS) based on Bray–Curtis dissimilarity, illustrating distinct clustering patterns among experimental groups. Statistical analyses were tested using ANOSIM. (**B**) Boxplots depicting distances of samples to group centroid are visualized. If one group has larger distances to the centroid than another, it suggests that microbial communities in that group are more heterogeneous, suggesting instability and dysbiosis. Statistical differences between groups were assessed by PERMDISP. (**C**) Alpha diversity, measured by the Shannon Index, demonstrating differences in species richness and evenness among groups. Only data with *p <* 0.05 were considered statistically significant (Table S2). Naive (n = 5), PBS (n = 15), Fh15 (n = 13), DSS (n = 15), and DSS-Fh15 (n = 15). Asterisks indicate significant differences between DSS and DSS-Fh15 groups only (* *p* = 0.03; *** *p* = 0.008).

To evaluate the potential impact of Fh15 on the gut microbiota composition of DSS mice, 16S rRNA gene sequencing was performed on fecal samples collected from all the experimental groups on days 2, 4, and 7. After quality assessment, a total of 63 samples were analyzed, with good quality reads of 13,053.646 ± 5481.723 (Table S1). The resulting data were used to analyze beta diversity, beta dispersion, and alpha diversity, providing insights into the overall community structure and diversity among treatment groups. DSS mice exhibited significantly different gut microbiota composition compared to both the control mice groups without colitis (naïve [ANOSIM *p* = 0.02] and PBS [ANOSIM *p* = 0.001]) and the UC-induced animals treated with Fh15 (ANOSIM *p* = 0.008), confirming the disruption of microbial communities during induced colitis (Figure 1A). Notably, colitic mice treated with Fh15 (DSS-Fh15) exhibited distinct microbial composition compared with the DSS group (ANOSIM, *p* = 0.046) (Figure 1A). Significant compositional differences were also observed when comparing the DSS-Fh15 group with the naive, PBS, and Fh15 groups (ANOSIM *p* < 0.001; Table S2), indicating that Fh15 treatment can partially modify the microbial composition induced by DSS but not to levels of healthy animals (Figure 1A). To assess the variability of microbial communities within groups, we performed beta-dispersion analysis, which showed no significant differences in dispersion among groups, except between the DSS and Fh15 groups (PERMDISP *p* = 0.032; Table S2; Figure 1B). However, the DSS-group showed greater distances from the group centroid compared to negative controls, suggesting increased microbial instability (Figure 1B). Fh15 treatment seems to have reduced this distance, with DSS-Fh15-treated mice showing shorter distances to the centroid than untreated DSS mice (Figure 1B). Shannon diversity remained constant in naive animals and those administered PBS or Fh15 (Figure 1C, Table S2). In contrast, DSS mice had significantly lower diversity compared to naive mice (KW *p =* 0.001). Moreover, with the pooled samples, across days, a significant difference was observed between the DSS and DSS-Fh15 groups (KW *p =* 0.036), indicating that Fh15 administration after colitis onset promotes a more diverse community (Figure 1C). However, major diversity differences were seen between negative control animals and DSS-Fh15-treated animals (KW *p <* 0.001), indicating that the changes induced by the Fh15 treatment are not enough to restore bacterial community diversity (Figure 1C).

### 2.2. Effect of Fh15 on the Gut Microbial Composition

**Figure 2:**
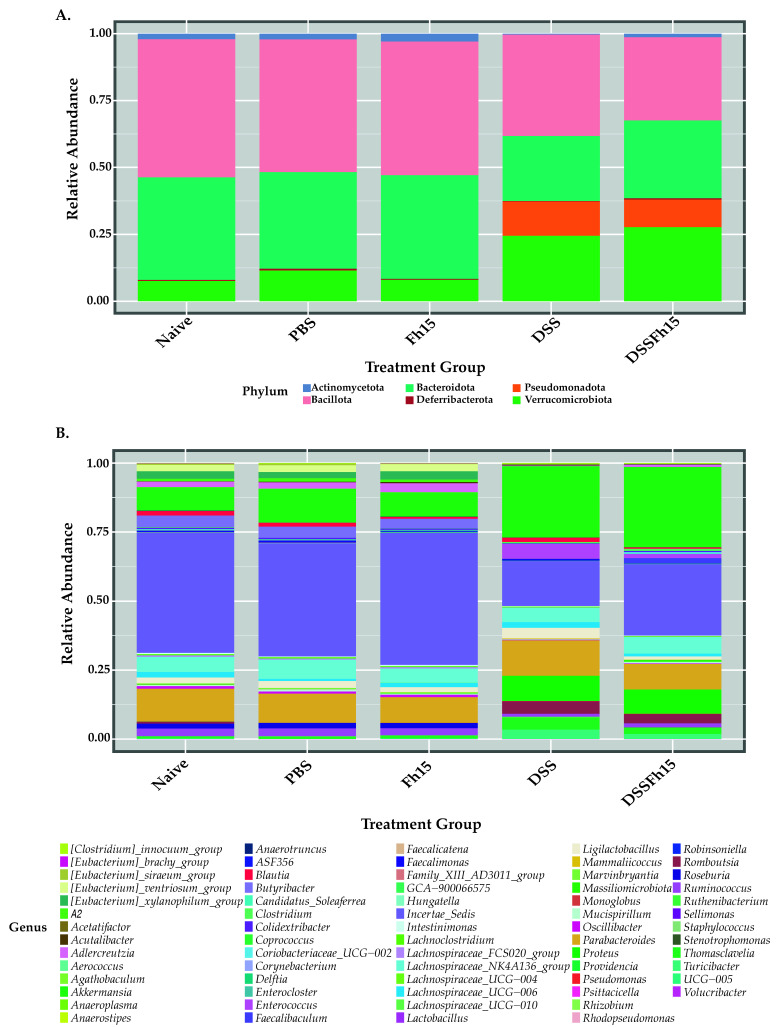
Fh15 shifts gut microbial taxa composition in DSS-induced ulcerative colitis mice. Taxonomic composition of gut microbiota at the (**A**) phylum and (**B**) genus levels across experimental groups (naive [n = 5], PBS [n = 15], Fh15 [n = 13], DSS [n = 15], and DSS-Fh15 [n = 15]). Relative abundances were calculated from 16S rRNA gene sequencing data and are represented as stacked bar plots, with colors indicating individual taxa.

To assess the impact of Fh15 on gut microbial composition, we employed the Bacillota/Bacteroidota (B/B) ratio as a key indicator of gut health to compare all samples across experimental groups (n = 63). The B/B ratio did not differ significantly between DSS and DSS-Fh15 or between DSS and control groups (Figure S1A). At the phylum level, LEfSe analysis identified distinct bacterial signatures among groups: Bacillota was the most significant in naive mice, Bacteroidota and Actinomycetota in Fh15 animals, Verrucomicrobiota in the DSS group, and Pseudomonadota in the DSS-Fh15 group (Figure S1B). Percentages represent differences in relative abundance between DSS and DSS-Fh15 groups. Taxonomic profiles showed that the DSS group exhibited a marked reduction in both Bacillota (−13.72%) and Bacteroidota (−14.04%) compared with naive mice (Figure 2A). At the genus level, biomarker signatures revealed that DSS mice had genera associated with inflammation and dysbiosis, including *Turicibacter, Enterococcus*, and *Proteus*, as well as other genera such as *Akkermansia*, *Romboutsia*, *Thomasclavelia*, *Ligilactobacillus*, and *Lachnospiraceae* (Figure S1C). In contrast, genera in non-colitic controls (naive, PBS, and Fh15) included several butyrate-producing and immunoregulatory taxa such as *Roseburia*, *Butyribacter*, *Adlercreutzia*, *Oscillibacter*, *Ruminococcus*, *Lachnospiraceae* UCG-006, and *Eubacterium*. Fh15 treatment in UC mice was associated with *Coriobacteriaceae* UCG-002, *Massiliomicrobiota*, *Clostridium*, and *Faecalibaculum* (Figure S1C). Genus-level taxonomic profiles revealed that *Akkermansia*, *Proteus*, *Enterococcus*, *Romboutsia*, and *Turicibacter* were enriched in DSS mice, while *Incertae sedis (unkown taxa)*, *Butyribacter*, and *Roseburia* were reduced or eliminated (Figure 2B). In contrast, Fh15 treatment partially recovered ~8.22% of taxa of unknown nomenclature in UC mice (Figure 2B). Together, these results suggest that Fh15 modulates the gut microbiota in UC mice, identifying potential bacterial signatures within treated and untreated animals.

### 2.3. Longitudinal Assessment of Fh15-Induced Modulation on Microbial Composition and Diversity in Ulcerative Colitis

**Figure 3:**
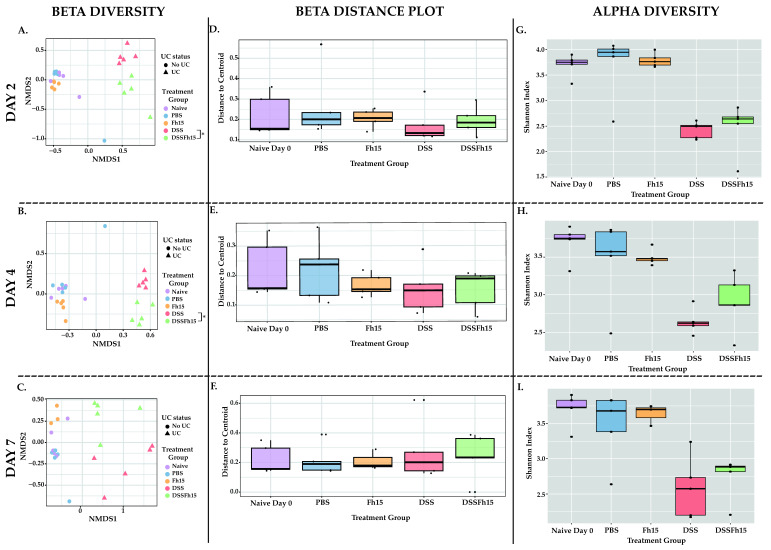
Fh15 treatment modulates gut microbial structure and restores community stability during early stages of ulcerative colitis. Non-metric multidimensional scaling (NMDS) beta diversity plots using the Bray–Curtis Dissimilarity Index matrix on (**A**) day 2, (**B**) day 4, and (**C**) day 7. Statistical significance between groups was assessed using ANOSIM. Beta dispersion boxplots illustrating within-group variability as distances from each sample to the group centroid on (**D**) day 2, (**E**) day 4, and (**F**) day 7. Alpha diversity measured by the Shannon Index across experimental groups to assess changes in microbial richness and evenness on (**G**) day 2, (**H**) day 4, and (**I**) day 7. Only data with *p <* 0.05 were considered statistically significant (Table S3). Each group contained five samples per day, except for the Fh15 group on day 7, which included only three samples. Asterisks indicate significant differences between DSS and DSS-Fh15 groups only (* *p* < 0.05).

To investigate the temporal dynamics of gut microbial changes during colitis and the modulatory effects of Fh15, we compared microbiota from naive animals on day 0 to those of animals sampled at day 2, day 4, and day 7 (Figure 3A–I). Each analysis included five animals per group per day (naive, PBS, DSS, DSS-Fh15), except for the Fh15 group on day 7, which included three animals. This design enabled assessment of baseline microbial communities relative to treatment conditions and timepoints, which allowed identification of temporally specific changes in microbial structure, distinguishing early onset (day 2) from later phases (days 4 and 7) of colitis progression. Bacterial community structure revealed significant differences when comparing control groups (PBS, Fh15, and naive) and UC mice (DSS and DSS-Fh15) (ANOSIM *p <* 0.05; Table S3). On day 2, the DSS and DSS-Fh15 groups showed significant compositional differences (ANOSIM *p =* 0.026; Table S3; Figure 3A), indicating that Fh15 rapidly modulated the microbiota during the initial phase of disease. By day 4, the divergence remained (ANOSIM *p =* 0.022; Table S3; Figure 3B), suggesting sustained microbial modulation as inflammation advanced. However, by day 7 the compositional differences between DSS and DSS-Fh15 were no longer statistically significant (ANOSIM *p =* 0.055; Table S3; Figure 3C), indicating that the modulatory effects of Fh15 are strongest during the early phase of colitis and diminish at later stages.

Bacterial dispersion was evaluated with Bray–Curtis distances per each individual timepoint (Figure 3D–F). On day 2, DSS mice exhibited significantly greater dispersion compared to naive (PERMDISP *p =* 0.042), PBS (PERMDISP *p =* 0.012), and Fh15 (PERMDISP *p =* 0.037) mice, reflecting increased community instability during early colitis induction (Table S3; Figure 3D). In contrast, dispersion in the DSS-Fh15 group was not significantly different from the control groups, suggesting that Fh15 helped maintain microbial heterogeneity at this early stage (PERMDISP *p* > 0.05; Table S3; Figure 3D). By day 4, DSS mice continued to show significantly higher dispersion relative to non-colitic controls, and the DSS-Fh15 group also began to diverge, showing increased dispersion compared to the naïve (PERMDISP *p =* 0.011) and PBS (PERMDISP *p =* 0.014) (Table S3; Figure 3E) groups. By day 7, microbial dispersion in DSS mice remained elevated relative to controls, while dispersion in the DSS-Fh15 group was no longer significantly different from that of non-colitic groups (PERMDISP *p* > 0.05), indicating potential community stability restoration at this later stage (Table S3; Figure 3F). Collectively, these findings suggest that Fh15 treatment mitigates DSS-induced microbial instability, with the strongest stabilizing effects apparent on days 2 and 7.

Alpha diversity was assessed using the Shannon Index to evaluate microbial richness and evenness across groups (Figure 3G–I). At all three timepoints, DSS mice exhibited a significant reduction in diversity compared to non-colitic controls (naive, PBS, and Fh15), confirming that colitis induction is associated with a loss of microbial diversity (KW *p <* 0.05; Table S3). For each timepoint, no significant diversity differences were observed between the DSS and DSS-Fh15 groups (KW *p* > 0.05; Figure 3G–I). These results confirm that DSS consistently disrupts overall microbial diversity during the early stages of acute colitis.

It is important to clarify that while pooled analysis across all timepoints revealed a significant difference between the DSS and DSS-Fh15 groups (KW *p* = 0.0362), timepoint-specific analyses did not detect significant differences on days 2, 4, or 7 individually. A limitation of this study is that intestinal barrier integrity was not directly assessed. Although our previous work using the same experimental model demonstrated preservation of epithelial architecture and reduced mucosal damage following Fh15 treatment [25], we did not perform functional permeability assays, such as FITC–dextran (FD-4), particularly at early time points (e.g., day 2) when the strongest microbiota effects were observed. Direct evaluation of epithelial permeability would provide important mechanistic insight into the early protective effects of Fh15 and should be addressed in future studies.

### 2.4. Temporal Genus-Level Microbial Changes

Genus-level taxonomic profiling of the top 25 most abundant taxa revealed microbial shifts across timepoints in response to DSS-induced colitis and Fh15 treatment in UC mice (Figure S2). On day 2, DSS led to a notable increase in the relative abundance of *Akkermansia*, *Ligilactobacillus*, *Parabacteroides*, and *Thomasclavelia*, while reducing *Blautia* in comparison with non-colitic groups (Figure S2). Additionally, several genera such as *Adlercreutzia*, *Eubacterium xylanophilum group*, *Butyribacter*, *Eubacterium ventriosum group*, *Roseburia*, and *Lachnospiraceae A2* were nearly or completely absent following DSS exposure. In contrast, colitic mice treated with Fh15 (DSS-Fh15) prevented loss of *Incertae sedis* (showing a 16.14% increase in abundance) and reduced the DSS-induced overgrowth of *Parabacteroides* (−10%), *Thomasclavelia* (−3.17%), and *Ligilactobacillus* (−3.68%). By day 4, microbial patterns remained roughly consistent with day 2 shifts, although a pronounced emergence of *Romboutsia* in the DSS group and an increase in *Turicibacter* in both the DSS and DSS-Fh15 groups was observed (Figure S2). By day 7, the abundances of *Blautia*, *Romboutsia*, *Turicibacter*, *Thomasclavelia*, and *Ligilactobacillus* increased in both the DSS and DSS-Fh15 groups compared to day 4, with consistently higher levels observed in the DSS group (Figure S2). Notably, pro-inflammatory taxa such as *Enterococcus* and *Proteus* appeared exclusively in the DSS and DSS-Fh15 groups on day 7, with a higher abundance in the DSS group (Figure S2). In addition, genera such *Akkermansia*, and *Parabacteroides* were significantly reduced by day 7 in the DSS group. In contrast, Fh15-treated UC mice maintained *Parabacteroides* levels comparable to the control groups, showed reduced abundances of *Enterococcus* (−8.53%) and *Turicibacter* (−3.64%) relative to DSS mice, and partially restored *Adlercreutzia* (+1.26%), the latter of which represents 1.91% abundance in naive mice. (Figure S2). 

### 2.5. DSS vs. DSS-Fh15 Longitudinal Sub-Cohort Analysis

**Figure 4:**
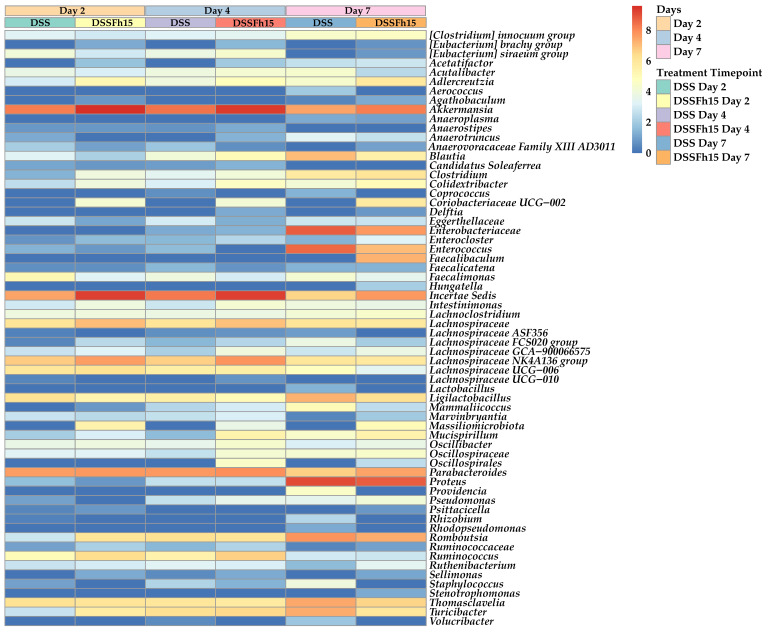
Genus-level heatmap across colitis progression of DSS and DSS-Fh15 groups. Heatmap comparison of relative abundances for bacterial genera in DSS (n = 14) and DSS-Fh15 (n = 14) groups on day 2, day 4, and day 7. The color scale ranges from blue, indicating lower values, to red, indicating higher values, with intermediate colors reflecting gradual increases across samples.

**Figure 5:**
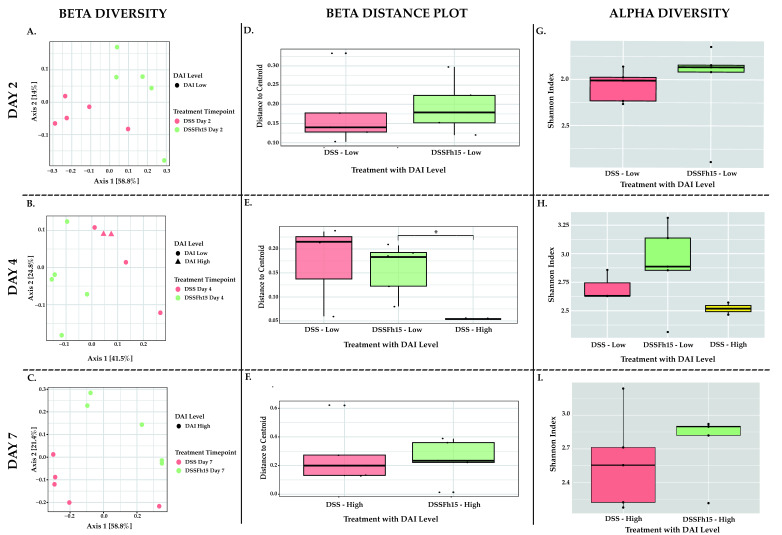
Diversity estimates and microbial structure in relation to disease activity index (DAI) during DSS-induced ulcerative colitis. Principal Coordinate Analysis (PCoA) using the Bray–Curtis Dissimilarity Index showing structural patterns between DSS and DSS-Fh15 mice on (**A**) day 2, (**B**) day 4, and (**C**) day 7, by disease activity index (DAI) levels. Beta dispersion boxplots illustrating variability in microbial community composition between DSS and DSS-Fh15 groups on (**D**) day 2, (**E**) day 4, and (**F**) day 7. Alpha diversity measured by the Shannon Index on (**G**) day 2, (**H**) day 4, and (**I**) day 7 in relation to DAI levels. Only data with *p <* 0.05 were considered statistically significant (Table S4). Day 2 (n = 10), day 4 (n = 10), and day 7 (n = 10). Asterisks indicate significant differences between DSS and DSS-Fh15 groups only (* *p* < 0.05).

A longitudinal sub-cohort microbial assessment was performed only for the DSS (n = 15) and DSS-Fh15 groups (n = 15) across the three timepoints. These numbers correspond to samples, not animals. For each timepoint (day 2, day 4, and day 7), the dataset included five samples of the DSS mice and five samples of the DSS-Fh15 group. Following quality assessment, 28 samples were retained for the joint microbial analysis of UC mice (Figure 4), whereas all 30 samples were included for the microbial assessment for the timepoint-specific characterization (Figure 5). This approach enabled direct bacterial dynamic comparisons between colitic untreated and Fh15-treated animals at equivalent timepoints.

We aimed to evaluate whether Fh15 could modulate or stabilize microbial communities during both the early onset (day 2) and progression (days 4 and 7) of DSS-induced colitis, thereby revealing treatment-specific effects on disease-associated microbial changes (Figure 4). In the DSS group, several beneficial genera such as *Adlercreutzia* declined progressively, particularly by day 7. In contrast, UC mice treated with Fh15 maintained higher relative abundance of these genera across all timepoints. Furthermore, genera associated with inflammation, such as *Enterococcus*, *Thomasclavelia*, and *Turicibacter*, were markedly elevated in DSS mice but remained lower in the DSS-Fh15 group, suggesting protective microbial modulation by Fh15 (Figure 4). These results highlight that Fh15 may help restore specific beneficial taxa and prevent higher expansion of potentially harmful bacteria during colitis.

Next, we incorporated previously calculated DAI levels [25] into the analysis of microbial communities. Bacterial community structure and composition on day 2 showed significant differences between DSS and DSS-Fh15 (PERMANOVA *p =* 0.031) (Table S4; Figure 5A). Although both groups showed the same DAI levels, their distinct clustering suggests that early Fh15 treatment helped preserve the microbial community independently of disease activity. By day 4, the DSS group displayed a mix of low and high DAI scores, while the DSS-Fh15 group maintained lower scores. Despite these differences, the two groups still clustered apart, with significant differences between the DSS–low-DAI-level group and the DSS-Fh15–low-DAI-level group (PERMANOVA *p =* 0.029), indicating that Fh15 continued to shape microbial structure while also maintaining reduced disease severity (Table S4; Figure 5B). On day 7, when all mice reached high DAI scores, the microbial communities of the DSS and DSS-Fh15 groups still clustered distinctly (PERMANOVA *p =* 0.053), with slightly reduced separation (Table S4; Figure 5C).

When comparing beta dispersion plots for each timepoint, there were no statistically significant differences on day 2, but we observed a trend in microbial community structure between DSS and DSS-Fh15 samples with low DAI levels (PERMDISP *p* > 0.05; Table S4; Figure 5D). By day 4, a significant difference in dispersion was observed between the DSS group, which displayed high DAI levels, and the DSS-Fh15 group, which maintained low DAI levels (PERMDISP *p =* 0.046; Table S4; Figure 5E). This suggests that Fh15 treatment not only maintained reduced disease severity but also stabilized microbial community structure, potentially preventing the dysbiosis associated with more severe inflammation (Figure 5E). On day 7, when both groups presented high DAI, microbial variability increased in all samples. However, DSS samples showed a different, but not significant, dispersion than DSS-Fh15 samples, suggesting that Fh15 partially changes the dysbiosis associated with UC even with higher inflammation levels (PERMDISP *p* > 0.05; Table S4; Figure 5F).

Although no statistically significant diversity differences were observed between groups at any timepoint, trends suggested that Fh15 helped preserve microbial diversity in UC mice (KW *p* > 0.05; Table S4; Figure 5G–I). On day 2, DSS samples showed reduced diversity compared to DSS-Fh15 mice even though they displayed the same low DAI level (Figure 5G). This finding suggests that Fh15 may help maintain microbial stability during the early phase of inflammation not correlated with disease severity. By day 4, when the DSS group exhibited low and high DAI scores, DSS-Fh15 mice maintained higher diversity, relative to DSS, consistent with the stabilization observed in beta diversity (KW *p* > 0.05; Table S4; Figure 5H). By day 7, at peak disease severity, diversity metrics decreased in both groups; however, DSS-Fh15 mice still exhibited modestly higher diversity than DSS mice (KW *p* > 0.05; Table S4; Figure 5I).

## 3. Discussion

Helminths and their excretory–secretory products have been shown to mitigate dysbiosis in experimental colitis by restoring microbial diversity and enhancing beneficial bacterial populations [26]. These parasites are well known for inducing strong Th2 and Treg immune responses and for reshaping the gut microbiota, often through the expansion of *Lactobacillus* species, commensals that themselves promote Tregs activity [27]. This reciprocal interaction establishes a helminth–microbiota relationship that promotes an immunoregulatory environment, supporting helminth persistence. Although helminths provide immunological benefits, they are considered pathogenic in humans and have been largely eliminated from modern settings, a shift linked to increased autoimmune diseases such as UC [28]. To mitigate the risks of live helminth infections, helminth-derived molecules are being explore as safer therapeutic alternatives that retain the immunomodulatory effects without pathogenic consequences [29]. Having previously shown that Fh15, a recombinant fatty acid-binding protein from *Fasciola hepatica*, markedly attenuates intestinal inflammation and reduces leukocyte infiltration in the colonic tissue of male mice with DSS-induced colitis [25], we sought to determine whether these therapeutic effects were linked to remodeling gut microbial communities. 

Beta diversity confirmed that DSS-induced colitis significantly altered gut microbial community structure compared to non-colitic controls, in agreement with previous studies demonstrating compositional changes during UC [30]. Fh15 treatment shifted bacterial communities of DSS mice toward profiles similar to those observed in non-colitic control groups, suggesting that protective taxa may have been retained. Timepoint-specific analyses revealed that Fh15-induced changes in gut microbiota composition were detectable as early as day 2 and appeared to peak by day 4, before declining on day 7. These results suggest that while Fh15 may exert a measurable modulatory effect when microbial re-stabilization is critical for disease outcomes [31,32], its influence is limited by the experimental design. Fh15 was administered intermittently while colitis was continuously induced with a relatively high DSS concentration. Consequently, Fh15 did not have sufficient time to counteract completely the rapid and aggressive dysbiosis caused by DSS exposure. However, as previously reported, given that by day 7 Fh15 treatment significantly reduced the disease activity index, multiple inflammatory markers (TNFα, IL-1β, MPO, CHI3L-1, and S100A9), and leukocyte infiltration [25], it is plausible that Fh15 enhances intestinal barrier integrity, thereby preventing bacterial translocation and attenuating colonic inflammation without markedly altering the dispersion or composition of luminal bacterial communities.

Community stability assessment highlighted that DSS treatment increased beta dispersion, reflecting heightened microbial heterogeneity and ecological imbalance typical of inflammation-induced dysbiosis [33]. In contrast, Fh15 treatment maintained microbial consistency early in the disease course and promoted recovery of stability by the later stages, suggesting a protective role in preserving ecological balance. Similar trends have been observed with helminth-based interventions, which stabilize microbial communities and mitigate inflammation-associated fluctuations [34]. Fh15 appeared to modulate the microbiota independently of disease severity. The treatment maintained a distinct microbial structure even as colitis progressed, highlighting its capacity to buffer against dysbiosis despite ongoing epithelial disruption. Elevated beta dispersion in colitis has been linked to a disruption in ecological balance [30], where inflammation causes random changes that disrupt the normal balance between the host and its gut microbiota, leading to a less predictable community composition [30]. Notably, DSS-Fh15 mice did not differ from non-colitic controls by day 2, suggesting that Fh15 preserved microbial consistency during early disease. By day 4, dispersion in DSS-Fh15 mice began to deviate from some controls. However, by day 7, dispersion levels in DSS-Fh15 mice were restored towards the non-colitic control group’s stability, whereas untreated DSS mice maintained an elevated dispersion. This pattern suggests that Fh15 reduces DSS-induced community instability at both early (day 2) and later (day 7) stages, potentially enhancing ecological balance. These results confirm that Fh15 exerts its strongest effects early in colitis independently of disease severity, aligning with evidence that helminth therapies help preserve microbiota balance and at the same time buffer against inflammation-associated dysbiosis [35]. These findings indicate that Fh15 primarily modulates microbial community composition and stability rather than overall species richness, consistent with its immunomodulatory function. Although colitic mice treated with Fh15 exhibited increased Shannon diversity compared with the DSS group, gut diversity was not fully restored to levels observed in non-colitic controls. The continuous administration of DSS likely counteracted some of the protective effects of Fh15, as persistent epithelial injury can repeatedly disrupt microbial homeostasis.

Microbial profiling revealed that DSS disrupted microbial equilibrium, reducing beneficial phyla such as Bacillota and Bacteroidota, as well as enriching Verrucomicrobiota, a pattern linked to mucin degradation [30,36,37]. Fh15 partially restored microbial balance by limiting the overgrowth of potentially proinflammatory genera (*Parabacteroides, Thomasclavelia,* and *Ligilactobacillus*) and reducing inflammation-associated taxa (*Enterococcus* and *Turicibacter*) [38,39]. Similarly, keeping the expansion of *Parabacteroides* and *Thomasclavelia* in check may prevent their pro-inflammatory potential [40], while limiting *Ligilactobacillus* overgrowth avoids excessive lactic acid accumulation that can destabilize gut ecology [41]. The reduction of *Enterococcus* and *Turicibacter* is also beneficial, as both are linked to heightened immune activation and worsened colitis severity [42,43,44]. Finally, the partial recovery of *Adlercreutzia* further suggests reinstatement of beneficial metabolic activity [45]. Together, these results indicate that Fh15 moderates the severity of dysbiosis by maintaining microbial stability and supporting the recovery of commensal populations associated with gut homeostasis. This microbial profile parallels restoration patterns seen in effective ulcerative colitis therapies, reinforcing Fh15’s potential as a helminth-derived biotherapeutic that combines anti-inflammatory and microbiota-modulating effects. We, however, need to acknowledge a major limitation of this study which is the relatively small sample size per experimental group, which may reduce statistical power to detect subtle microbiota shifts.

Although these findings do not establish a direct causal relationship between Fh15 and the observed microbial shifts, the anti-inflammatory properties of Fh15 observed in our previous study provide a plausible explanation [25]. By reducing colonic leukocyte infiltration and limiting the activation of CD11b^+^ CD11c^−^ CD86^+^ myeloid cells, Fh15 decreases pro-inflammatory cytokine levels and alleviates luminal stress [25]. This could decrease host electron acceptors (e.g., oxygen and nitrate) that favor the overgrowth of facultative opportunistic pathogens, while restoring the anaerobic conditions preferred by SCFA-producing Bacillota/Bacteroidota [30,46,47]. In parallel, Fh15’s epithelial protective effects, which include reducing CHI3L1 expression, a host factor involved in mucus–glycan interactions, would diminish mucus and host–glycan disruption, thereby helping preserve the mucus-associated niche and further stabilizing community structure [48], supporting the persistence of beneficial taxa. However, we hypothesize that these effects are likely indirect, as intraperitoneal administration of Fh15 leads to its localization in the spleen, a key site of immune activation, where it remains detectable up to 24 hours post-administration [23].

Fh15 has demonstrated significant suppression of inflammatory markers and a reduction in disease activity index [25]. However, the observed effects on the gut microbiota were less pronounced than anticipated. The use of an acute colitis model induced by 4% DSS, coupled with the intermittent administration of Fh15, likely constrained the full assessment of its microbial modulatory potential. Helminth infections are typically chronic, allowing continuous host–parasite interactions that promote long-term immune adaptation. Helminth-derived molecules, such as Fh15, exert their immunomodulatory effects primarily through the induction of a Th2- and Treg-biased response, which collectively suppresses excessive inflammation and promotes tissue homeostasis and immune homeostatic tolerance [49]. When exposure to helminth-derived molecules occurs prophylactically, before the onset of intestinal inflammation, a more balanced immune environment can develop, potentially preventing or attenuating the progression of colitis more effectively than therapeutic intervention during active disease. This concept is particularly relevant to clinical settings, as patients with inflammatory bowel disease experience heightened systemic inflammation and an increased risk of venous thromboembolism (VTE) driven by elevated circulating pro-inflammatory cytokines. The risk of VTE escalates during disease flares, underscoring the importance of prophylactic management in acute episodes [50]. It is clear that both in preclinical models as in the clinical setting, timely and targeted modulation of the immune response and the microbiome, eventually through prophylactic use of immunoregulatory molecules like Fh15, may not only improve intestinal outcomes but also mitigate systemic inflammatory complications. Future studies evaluating coagulation markers and thrombotic risk will be necessary to determine whether Fh15-mediated microbiota modulation has systemic effects beyond intestinal inflammation. The concept of early immune regulation is supported by multiple studies using DSS-induced colitis models, which demonstrate that helminth-derived products show greater efficacy when administered preventively rather than reactively [51,52]. Therefore, while Fh15 exhibits measurable anti-inflammatory and microbiota-stabilizing properties, its full therapeutic potential may depend on the timing and mode of administration. Future studies employing prophylactic treatment regimens or chronic colitis models may better elucidate the extent of Fh15’s immunoregulatory and microbiota-modulating capabilities.

## 4. Materials and Methods

### 4.1. Animals and Ethics Statement

Twenty-five male C57BL/6 mice (6–8 weeks old) were purchased from Charles River Laboratories (Wilmington, MA, USA). Animals were maintained under standard laboratory conditions at 21 °C with a 12 h light/dark cycle and provided ad libitum access to food and water. Mice were housed in a controlled environment with monitored temperature and humidity to ensure stable housing conditions. Following an acclimatization period, animals were used for experimental procedures. Each mouse represented an experimental unit (n = 5/group) and was sampled longitudinally at three time points (days 2, 4, and 7), generating multiple samples per animal. After sequencing and quality filtering, samples with low read counts were excluded according to predefined quality criteria, and 63 samples were retained for downstream analysis. All experimental procedures were conducted in accordance with institutional ethical guidelines and were approved by the Institutional Animal Care and Use Committee (IACUC; Protocol #7870123) and the Biosafety Committee (Protocol #IBC156523) of the University of Puerto Rico Medical Sciences Campus.

### 4.2. Recombinant Fasciola hepatica FABP (Fh15)

Recombinant Fh15 was expressed in *Bacillus subtilis* and purified endotoxin-free following a pre-established protocol [24]. The batch used in the present study had a protein concentration of 2.29 mg/mL, endotoxin levels below 0.4 EU/mg and a purity greater than 90%, as confirmed by blue staining densitometry and LC–MS/MS [24].

### 4.3. Fh15 Treatment Administration, Dextran Sulfate Sodium (DSS) Colitis Induction, and Fecal Sample Collection

Fecal samples analyzed in this study were collected from mice subjected to DSS-induced ulcerative colitis in a previous experiment assessing the therapeutic potential of Fh15 to reduce intestinal inflammation [25]. Mice were randomly assigned to five experimental groups (naive, PBS, Fh15, DSS [colitic–non-treated], and DSS-Fh15 [colitic–treated]), with five animals per group. Groups sample size was selected based on prior experience and comparable studies in the field, balancing the ability to detect biologically meaningful differences with the ethical principle of minimizing animal use. Investigators were aware of group allocation during the conduct of the experiment and data analysis. Naive mice served as untreated controls and received standard drinking water. The PBS control group received regular water and intraperitoneal (i.p.) injections of 50 µL endotoxin-free phosphate-buffered saline (PBS; 0.1 M, pH 7.2; Gibco, Grand Island, NY, USA) on days 1, 3, and 5. The Fh15 control group received the same injection schedule with Fh15 (2.0 mg/kg body weight) diluted in PBS. Colitis was induced in the DSS and DSS-Fh15 groups by providing 4% (*w*/*v*) DSS (40 kDa; Sigma-Aldrich, Burlington, MA, USA) in autoclaved drinking water ad libitum for seven days, as described by Chassaing et al. (2014) [52]. DSS concentration was selected to induce a severe and reproducible colitis phenotype in C57BL/6 mice, allowing for a rigorous assessment of Fh15’s anti-inflammatory effects under high inflammatory burden. The DSS-Fh15 group also received Fh15 i.p. injections (2.0 mg/kg) on days 1, 3, and 5 of DSS treatment. Fecal pellets were collected individually from each mouse on days 2, 4, and 7 of treatment, coinciding with the day following each i.p. injection (n = 65). Fh15 dosing regimen was selected to maintain sustained immunomodulatory activity throughout disease progression, as repeated administration of helminth-derived molecules is commonly required to achieve consistent regulation of host immune responses and microbiota dynamics [53]. The order of sample collection and measurements was randomized to minimize potential order effects. Samples were stored at −80 °C until use. Body weight data and related calculations for this cohort have been extensively reported in a companion study examining the effects of Fh15 on colonic inflammation and leukocyte infiltration in DSS-induced colitis [25].

### 4.4. Disease Activity Index (DAI) Classification

Mice were monitored daily for changes in body weight, stool consistency, and hematochezia. Stool consistency was evaluated macroscopically using the following scale of four scores: 0 (formed and hard), 1 (formed but soft), 2 (loose stool), and 3 (watery). Presence of blood in stool was evaluated by a four-score scale: 0 (no bleeding), 1 (positive hemoccult (Beckman Coulter, Brea, CA, USA)/no visible blood), 2 (visible blood in stool), and 3 (fresh rectal bleeding). Finally, weight loss clinical score was assigned as follows: 0 (<2%), 1 (≥2%–<5%), 2 (≥5%–<10%), 3 (≥10%–<15%), or 4 (>15%). The summed scores yielded a total DAI ranging from 0 to 10, with higher scores indicating greater disease severity. DAI values were stratified into two severity levels: low (0–5 DAI) and high (6–10 DAI). This classification was implemented as a variable in the metadata table using a conditional formula in Microsoft Excel 365 v.16.108.2 (Microsoft Corporation, Redmond, WA, USA) to ensure consistent grouping of samples for downstream analysis. These disease index parameters have been previously used by our group [25].

### 4.5. Genomic DNA Extraction and 16S rRNA Gene Sequencing

Genomic DNA (gDNA) was extracted from fecal pellets collected from C57BL/6 male mice on days 2, 4, and 7, corresponding to post-Fh15 administration time points, with day 7 representing the experimental endpoint, to capture early, intermediate, and late-stage microbiota dynamics during disease progression. DNA extraction was performed using the DNeasy PowerSoil Pro Kit (QIAGEN, Germantown, MD, USA), following the manufacturer’s protocol with minor modifications. Briefly, (1) an equal volume of EA solution and 100% ethanol was mixed with the supernatant; (2) lysates were passed through spin filter columns using a vacuum manifold; (3) prior to adding solution CD5, the column was washed with 650 µL of 100% ethanol (Spectrum Chemical Manufacturing Corp., New Brunswick, NJ, USA); and (4) gDNA was eluted with 100 µL of pre-warmed (55 °C) solution C6. As prior DNA libraries yield few to no reads (low yield) and to ensure removal of PCR inhibitors, particularly residual DSS, the extracted DNA from all samples was purified using the ZymoBIOMICS DNA Miniprep Kit (Zymo Research, Irvine, CA, USA). DNA concentrations were measured using the Qubit 1X dsDNA HS Assay Kit (Thermo Fisher Scientific, Waltham, MA, USA) and the Qubit 2.0 Fluorometer. DNA samples were stored at −20 °C until they were sent to an outsourced laboratory for sequencing. Remaining DNA samples were kept at −80 °C for long-term storage.

Amplification of the 16S rRNA hypervariable region 4 (V4) was performed using the universal primers 515F (5′-GTGCCAGCMGCCGCGGTAA-3′) and 806R (5′-GGACTACHVGGGTWTCTAAT-3′), following the Earth Microbiome Project standard protocols (https://earthmicrobiome.org/protocols-and-standards/ (accessed on 13 March 2026)). Amplicons were sequenced on the Illumina MiSeq platform using a 2 × 250 base paired-end protocol.

### 4.6. Pre-Processing and Quality Control

Raw 16S rRNA sequences were demultiplexed and pre-processed using the QIITA platform (https://qiita.ucsd.edu/ (accessed on 13 March 2026); Knight Lab, University of California San Diego, La Jolla, CA, USA) with a default Phred score offset of 30 [54]. Sequences were trimmed to 250 base pairs (bp) followed by a denoising workflow using the Deblur algorithm (Deblur 2021.09). Taxonomy assignment of the amplicon sequence variants (ASVs) was done with the SILVA reference database (v.138.2, SILVA Team, Bremen, Germany) at a 97% similarity threshold [55,56].

The resulting feature table was downloaded from QIITA for further downstream processing. Singletons, mitochondria, and chloroplast sequences were removed prior to analyses. Microbial community analyses, including beta diversity, Shannon Index diversity metric [57], and taxonomic composition, were performed using QIIME2 (qiime2-amplicon-2024.10, Flagstaff, AZ, USA) [58]. To account for differences in sequencing depth, rarefaction was applied. Note that different analysis groups had different read depths depending on the sample groupings (individual analyses) (Table S1).

The dataset and corresponding metadata are publicly available through the QIITA platform under study ID 15768 as well as in the European Nucleotide Archive (ENA) through the accession number ERP185110. The metadata variables analyzed in this study were treatment group, treatment time point, and disease activity index level.

### 4.7. Beta Diversity

The composition and structure of bacterial communities across experimental groups were assessed using the Bray–Curtis Dissimilarity Index [59,60] and, in some cases, Principal Coordinate Analysis (PCoA) [61]. While non-metric multidimensional scaling (NMDS) [62] preserves only the relative rank order of distances in a reduced-dimensional space, PCoA provides a linear representation of the distances [63]. Beta diversity plots were visualized using the phyloseq [64] and ggplot2 [65] R (v.4.3.2., R Foundation for Statistical Computing, Vienna, Austria) packages. Statistical significance among groups was evaluated using the Permutational Multivariate Analysis of Variance (PERMANOVA) [66,67] and Analysis of Similarities (ANOSIM) [68] applied to the PCoA and NMDS plots, respectively. To ensure robustness, PERMANOVA tests were paired with a Permutational Multivariate Analysis of Dispersion (PERMDISP) [69], which compares the spread or variability among groups. In addition, PCoA plots paired with the PERMANOVA test were specifically used to compare DSS and DSS-Fh15 samples, based on filtered feature tables with data collected on day 2 (n = 10), day 4 (n = 8), and day 7 (n = 10).

### 4.8. Alpha Diversity

Alpha diversity metrics were employed to evaluate richness and diversity within the bacterial communities. The Shannon Index was calculated to estimate sample richness and evenness [57]. To compare pairwise microbiota diversity among groups of animals, we employed the non-parametric Kruskal–Wallis (KW) pairwise test [70].

### 4.9. Taxonomic Abundance Profiles

Bacterial taxonomic profiles were summarized at the phylum and genus levels. Relative abundance tables were generated in QIIME2 and exported for further visualization. Figures were constructed in R using the phyloseq [64], vegan [71], and ggplot2 [65] packages. Additional figures were also generated to show the top 25 most abundant taxa at th genus level. For the feature table containing only the DSS and DSS-Fh15 samples across the three timepoints (n = 28), genus-level counts underwent normalization with the DESeq2 package [72], and a heatmap was created using the dplyr [73] and pheatmap [74] packages.

### 4.10. Bacillota/Bacteroidota Ratio

Given that Bacillota and Bacteroidota constitute most of the mammalian gut microbiota, the Bacillota/Bacteroidota (B/B) ratio (previously known as the Firmicutes/Bacteroidetes ratio) was calculated as a potential indicator of microbial dysbiosis. Count abundance values at the phylum level were used to compute the B/B ratio for each sample. Visualization was performed in R with the vegan package [71]. To evaluate statistical significance between groups, the Wilcoxon Rank-Sum Test (WRST) [75] was applied, with significance thresholds set at *p*-value < 0.05. This is a commonly reported but debated indicator of microbial imbalance.

### 4.11. Microbial Biomarkers

To identify potential microbial biomarkers associated with DSS and Fh15 treatment outcomes, we applied a Linear Discriminant Analysis (LDA) Effect Size (LEfSe) [76] using the microeco [77] and ggplot2 [65] R packages. This method combines a non-parametric KW test to determine differentially abundant taxa among groups with an LDA score to estimate the effect size of each feature. Taxa with an LDA score of 2.0 and a *p*-value < 0.05 were considered significant and biologically relevant.

## 5. Conclusions

This study demonstrates that Fh15 modulates the gut microbiota during DSS-induced colitis, exerting its strongest effects during the early stages of disease. Fh15 treatment shifted community structure toward that of non-colitic controls, reduced microbial dispersion, indicating enhanced community stability, and partially preserved microbial diversity. In addition, Fh15 limited the expansion of inflammation-associated taxa and supported the persistence of beneficial genera, consistent with its anti-inflammatory and epithelial-protective properties [25]. Together, these findings suggest that Fh15 mitigates inflammation-associated microbial dysbiosis and contributes to maintaining a more stable gut ecosystem during severe colitis progression.

However, several limitations should be considered. First, the use of an acute 4% DSS model, combined with intermittent Fh15 administration, likely constrained the ability of the treatment to fully counteract the rapid and severe dysbiosis and epithelial injury characteristic of this model. Second, although microbiota dynamics were assessed longitudinally, the relatively small sample size per group may have limited statistical power to detect subtle but biologically relevant changes. Third, intestinal barrier integrity was not directly evaluated. While our previous study using the same experimental model demonstrated preservation of epithelial architecture and reduced mucosal damage following Fh15 treatment, functional permeability assays (e.g., FITC–dextran) were not performed, particularly at early time points such as day 2, when the strongest microbiota effects were observed. Finally, systemic outcomes related to coagulation or thrombotic risk were not assessed, and therefore any link between microbiota modulation and thrombosis remains indirect and speculative. These preliminary findings highlight the potential of helminth-derived molecules, such as Fh15, as safer therapeutic alternatives that replicate the immunoregulatory and microbiota-modulating effects of live helminth infections without their associated risks.

Importantly, the data suggest that prophylactic or sustained administration may enhance efficacy by establishing an anti-inflammatory and microbiota-stabilizing environment before disease onset. Future studies using chronic or preventive treatment models, incorporating direct assessment of epithelial barrier function and systemic parameters, will be essential to fully define the therapeutic potential of Fh15 and its capacity to mitigate both intestinal inflammation and extraintestinal complications.

## Data Availability

All data are presented in this manuscript and provided as Supplementary Information. 16S rRNA gene sequences can be found in the QIITA study #15768 (sandbox ID 18469) (https://qiita.ucsd.edu/study/description/15768# (accessed on 13 March 2026)). They are also available in the European Nucleotide Archive ENA Project ERP185110.

## Supplementary Materials

The following supporting information can be downloaded at: https://www.mdpi.com/article/10.3390/ijms27094068/s1.
