# Supplementary material for: Intermittent Administration of Helminth-Derived Fh15 Modulates Gut Microbiota and Partially Mitigates Dysbiosis in Early Stages of Severe Experimental Colitis
Source: Int J Mol Sci. 2026 May 2;27(9):4068. doi: 10.3390/ijms27094068 (PMC13164252; doi:10.3390/ijms27094068)
Supplement: Supplementary file 1 [file ijms-27-04068-s001.zip › ijms-4228697-supplementary.pdf]

SUPLEMENTARY MATERIAL

Table S1. Sample distribution and rarefaction depths applied per feature table for downstream microbiome analyses.

Feature table	Samples per feature table	Group	Samples per group	Timepoint	Reads (Average ± Stdev)	Rarefaction
**1**	63	Naive	5	Day 0	13053.646 ± 5481.723	4582
		PBS	13	Day 2, Day 4, and Day 7		
		Fh15	13	Day 2, Day 4, and Day 7		
		DSS	15	Day 2, Day 4, and Day 7		
		DSS-Fh15	15	Day 2, Day 4, and Day 7		
**2**	25	Naive	5	Day 0	11756.880 ± 4654.080	4582
		PBS	5	Day 2		
		Fh15	5	Day 2		
		DSS	5	Day 2		
		DSS-Fh15	5	Day 2		
**3**	25	Naive	5	Day 0	13269.680 ± 6015.561	6540
		PBS	5	Day 4		
		Fh15	5	Day 4		
		DSS	5	Day 4		
		DSS-Fh15	5	Day 4		
**4**	23	Naive	5	Day 0	13278.130 ± 3981.181	6540
		PBS	5	Day 7		
		Fh15	3	Day 7		
		DSS	5	Day 7		
		DSS-Fh15	5	Day 7		
**5**	28	DSS	14	Day 2, Day 4, and Day 7	13,723.679 ± 3,955.920	4582
		DSS-Fh15	14	Day 2, Day 4, and Day 7		
**6**	10	DSS	5	Day 2	13368.700 ± 4780.502	4579
		DSS-Fh15	5	Day 2		
**7**	10	DSS	5	Day 4	13968.000 ± 4770.745	8445
		DSS-Fh15	5	Day 4		
**8**	10	DSS	5	Day 7	14036.700 ± 2202.314	10906
		DSS-Fh15	5	Day 7		

Table S2. Statistical tests corresponding to Figure 1.

			16S rRNA gene		
			Figure 1A	Figure 1B	Figure 1C
	**Experimental Group 1**	**Experimental Group 2**	**ANOSIM p-value**	**PERMDISP p-value**	**Kruskal-Wallis p-value**
**DSS-Induced Colitis Experiment**	Naive	PBS	0.481	0.497	0.965189
	Naive	Fh15	0.071	0.985	0.459727
	Naive	DSS	**0.022**	0.178	**0.001063**
	Naive	DSS-Fh15	**0.001**	0.275	**0.001443**
	PBS	Fh15	**0.001**	0.202	0.628609
	PBS	DSS	**0.001**	0.100	**0.000147**
	PBS	DSS-Fh15	**0.001**	0.333	**0.001130**
	Fh15	DSS	**0.001**	**0.032**	**0.000007**
	Fh15	DSS-Fh15	**0.001**	0.100	**0.000007**
	DSS	DSS-Fh15	**0.008**	0.396	**0.036203**

***Significant p-values in bold.**

Table S3. Statistical tests corresponding to Figure 3.

			16S rRNA gene								
			Day 2			Day 4			Day 7		
			Figure 3A	Figure 3D	Figure 3G	Figure 3B	Figure 3E	Figure 3H	Figure 3C	Figure 3F	Figure 3I
	**Experimental Group 1**	**Experimental Group 2**	**ANOSIM p-value**	**PERMDISP p-value**	**KW p-value**	**ANOSIM p-value**	**PERMDISP p-value**	**KW p-value**	**ANOSIM p-value**	**PERMDISP p-value**	**KW p-value**
**DSS-Induced Colitis Experiment**	Naive Day 0	PBS	0.339	0.604	0.175	0.330	0.948	0.602	0.207	0.896	0.347
	Naive Day 0	Fh15	0.407	0.716	0.754	0.413	0.168	0.117	0.316	0.883	0.456
	Naive Day 0	DSS	**0.012**	**0.042**	**0.009**	**0.006**	**0.011**	**0.009**	**0.010**	**0.006**	**0.009**
	Naive Day 0	DSS-Fh15	**0.007**	0.368	**0.009**	**0.013**	**0.011**	**0.016**	**0.011**	0.388	**0.009**
	PBS	Fh15	**0.013**	0.602	0.251	**0.021**	0.323	0.251	0.054	0.807	0.881
	PBS	DSS	**0.007**	**0.012**	**0.016**	**0.005**	0.103	0.076	**0.013**	**0.014**	**0.028**
	PBS	DSS-Fh15	**0.010**	0.061	**0.047**	**0.009**	**0.014**	0.076	**0.009**	0.361	0.076
	Fh15	DSS	**0.009**	**0.037**	**0.009**	**0.008**	0.511	**0.009**	**0.025**	0.257	**0.025**
	Fh15	DSS-Fh15	**0.012**	0.551	**0.009**	**0.011**	0.167	**0.009**	**0.018**	0.438	**0.025**
	DSS	DSS-Fh15	**0.026**	0.589	0.175	**0.022**	0.991	0.251	0.055	0.688	0.251

***Significant p-values in bold.**

Table S4. Statistical tests corresponding to Figure 5.

			16S rRNA gene								
			Day 2			Day 4			Day 7		
			Figure 5A	Figure 5D	Figure 5G	Figure 5B	Figure 5E	Figure 5H	Figure 5C	Figure 5F	Figure 5I
	**Experimental Group 1**	**Experimental Group 2**	**PERMANOVA p-value**	**PERMDISP p-value**	**KW p-value**	**PERMANOVA p-value**	**PERMDISP p-value**	**KW p-value**	**PERMANOVA p-value**	**PERMDISP p-value**	**KW p-value**
**DSS-Induced Colitis Experiment**	DSS (low DAI)	DSS-Fh15 (low DAI)	**0.031**	0.610	0.251	**0.029**	0.847	0.480	-	-	-
	DSS (high DAI)	DSS (low DAI)	-	-	-	0.609	0.198	0.083	-	-	-
	DSS (high DAI)	DSS-Fh15 (low DAI)	-	-	-	0.086	**0.046**	0.355	-	-	-
	DSS (high DAI)	DSS-Fh15 (high DAI)	-	-	-	-	-	-	0.053	0.701	0.347

***Significant p-values in bold.**

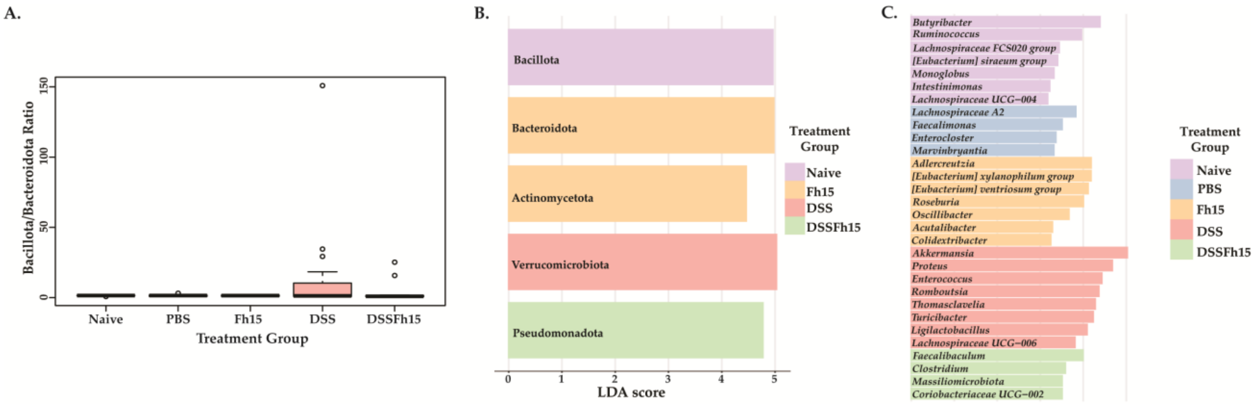

Figure S1. **Comparative analysis of gut microbiota composition and differential taxa abundance in DSS-induced ulcerative colitis mice treated with Fh15.** (**A**) Bacillota/Bacteroidota (B/B) ratio, calculated from phylum level abundance data for each group. To evaluate statistical significance between groups, the Wilcoxon Rank-Sum Test (WRST) was applied, with significance thresholds set at *p*-value<0.05. (**B**) Identification of biomarkers using Linear Discriminant Analysis Effect Size (LEfSe) combined with a non-parametric Kruskal-Wallis (KW) test at the phylum level. (**C**) Identification of biomarkers using Linear Discriminant Analysis Effect Size (LEfSe) combined with a non-parametric Kruskal-Wallis (KW) test at the genus level. Taxa with an LDA score of 2.0 and a *p*-value<0.05 were considered significant. Naive (n=5), PBS (n=15), Fh15 (n=13), DSS (n=15), and DSS-Fh15 (n=15).

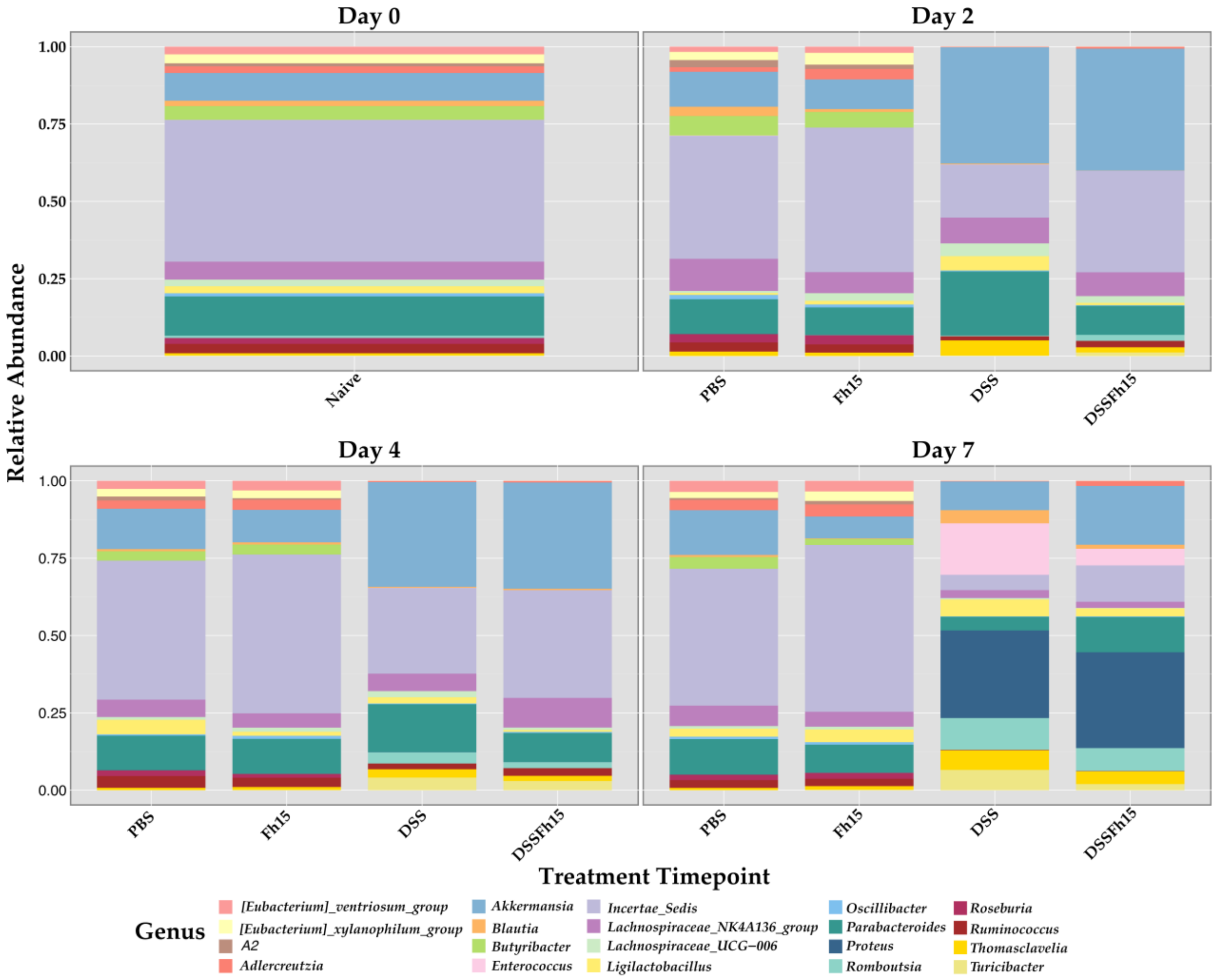

Figure S2. Gut microbial taxonomic abundance changes at genus level in DSS-induced ulcerative colitis mice treated with Fh15. Relative abundance of the top 25 bacterial genera across experimental groups (PBS [n=15], Fh15 [n=13], DSS [n=15], and DSS-Fh15 [n=15]) at day 2, day 4, and day 7, including naive group at day 0 (n=5).
